# Generative Artificial Intelligence and Large Language Models in Clinical Oncology

**DOI:** 10.1002/mco2.70833

**Published:** 2026-06-23

**Authors:** Yunfang Yu, Zhenhui Zhao, Zehua Wang, Ruichong Lin, Yujie Tan, Yongjian Chen, Ting Li, Daniel Baptista‐Hon, Xiaoxi Zhang, Chuan Wu, Man Tong, Lijun Zheng, Junyan Wu, Olivia Monteiro, Kang Zhang

**Affiliations:** ^1^ Artificial Intelligence Cross Disciplinary Research Institute, Faculty of Medicine, Faculty of Innovation Engineering, School of Computer Science and Engineering Macau University of Science and Technology Macau SAR China; ^2^ Department of Medical Oncology, Department of Pharmacy, Phase I Clinical Trial Centre, Sun Yat‐sen Memorial Hospital, School of Computer Science and Engineering & Key Laboratory of Machine Intelligence and Advanced Computing Sun Yat‐sen University Guangzhou China; ^3^ Department of Breast Surgery, The First Affiliated Hospital Jinan University Guangzhou China; ^4^ Guangdong Provincial Key Laboratory of Cancer Pathogenesis and Precision Diagnosis and Treatment, AI Big Data Laboratory, Shenshan Medical Center Memorial Hospital of Sun Yat‐Sen University Shanwei China; ^5^ Department of Medicine Solna, Center For Molecular Medicine Karolinska Institutet Stockholm Sweden; ^6^ School of Medicine University of Dundee Dundee UK; ^7^ Shenzhen Loop Area Institute Shenzhen China; ^8^ School of Computing and Data Science The University of Hong Kong Hong Kong SAR China; ^9^ School of Biomedical Sciences The Chinese University of Hong Kong Hong Kong SAR China; ^10^ China United Network Communications Corporation GuangZhou Branch Guangzhou China; ^11^ The First Affiliated Hospital of Chongqing Medical University Chongqing China; ^12^ Department of Ophthalmology, Zhuhai People's Hospital and the First Affiliated Hospital of Faculty of Medicine Macau University of Science and Technology Zhuhai China; ^13^ Guangzhou National Laboratory Guangzhou China; ^14^ Eye and Vision Innovation Center Eye Valley Wenzhou China

**Keywords:** clinical decision support, generative artificial intelligence, large language models, multimodal data integration, oncology

## Abstract

Cancer remains a major global health challenge, and the increasing availability of multimodal biomedical data has created unprecedented opportunities for precision oncology. Recent advances in generative artificial intelligence (AI), particularly large language models (LLMs), have enabled new approaches for integrating heterogeneous data sources, including electronic health records, medical imaging, pathology, genomics, and clinical text. However, current studies remain fragmented across specific tasks, cancer types, and model architectures, and a comprehensive synthesis of how generative AI can support the entire oncology continuum is still lacking. This review provides an overview of generative AI in clinical oncology, covering LLMs, generative adversarial networks, diffusion models, and multimodal foundation models. We summarize their methodological foundations and discuss applications in cancer diagnosis, prognosis prediction, treatment planning, patient management, and clinical trial optimization. Particular attention is given to multimodal data integration, synthetic data generation, clinical reasoning, and decision support, together with current challenges related to interpretability, reliability, data privacy, regulatory governance, and real‐world implementation. By consolidating recent technological advances and clinical evidence, this review highlights future priorities toward safe, trustworthy, and clinically deployable intelligent oncology systems. Emerging agent‐based architectures and human–AI collaborative workflows may further expand the clinical utility of generative AI in oncology.

## Graphical abstract

By integrating multimodal data, including medical imaging, pathology, omics, and electronic health records, generative AI and LLMs support cancer diagnosis, treatment planning, and follow‐up management. These technologies also enhance physician–patient communication, improve personalized treatment strategies, and leverage intelligent agent automation to support more accurate clinical decision‐making and real‐time disease management.

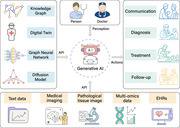

## 1 Introduction

Cancer remains a leading cause of global disease burden and mortality, with escalating impact driven by population aging, lifestyle changes, and environmental exposures [1, 2, 3, 4]. The 2022 Global Cancer Statistics report documented approximately 20 million new cancer cases and 9.7 million cancer‐related deaths worldwide, and this number is projected to increase substantially over the coming decades [5]. Precision oncology has emerged as a key strategy for addressing this challenge through personalized prevention, diagnosis, and treatment [6]. Advances in high‐throughput sequencing, multiomics technologies, digital pathology, and medical imaging have generated unprecedented volumes of heterogeneous biomedical data [7]. However, effectively integrating these multimodal data into clinically actionable knowledge remains a major challenge in modern cancer care.

Artificial intelligence (AI) has increasingly transformed oncology by improving data interpretation and clinical decision‐making [8, 9, 10, 11]. Conventional deep learning approaches have achieved remarkable success in tumor detection, histopathological classification, radiomics analysis, and outcome prediction [12, 13, 14, 15]. More recently, generative AI has emerged as a new paradigm capable of learning complex biomedical representations and generating clinically meaningful outputs. This rapidly evolving field encompasses large language models (LLMs), generative adversarial networks (GANs), diffusion models, vision–language models, and multimodal foundation models [16, 17, 18, 19, 20]. Beyond conventional predictive tasks, generative AI can synthesize medical images, generate clinical reports, augment scarce datasets, model molecular structures, and facilitate multimodal reasoning, providing novel opportunities for precision oncology [21, 22, 23, 24, 25].

**FIGURE 1:**
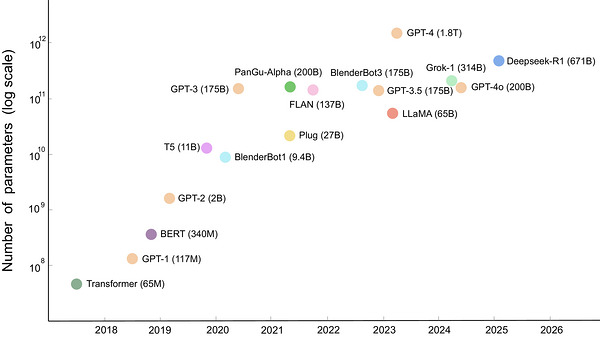
Scaling trajectory of LLMs from 2017 to 2026. The plot illustrates the evolution of representative large language models, with parameter counts shown on a logarithmic scale. Early architectures, including transformer (65 M), GPT‐1 (117 M), and BERT (340 M), established the foundation for pretrained language modeling. This was followed by a rapid transition to large‐scale models such as GPT‐2 (2B), T5 (11B), and GPT‐3 (175B). More recent models—including FLAN (137B), PanGu‐Alpha (200B), LLaMA (65B), GPT‐3.5 (175B), GPT‐4 (∼1.8T), GPT‐4o (200B), Grok‐1 (314B), and DeepSeek‐R1 (671B)—demonstrate continued expansion in parameter scale alongside architectural refinement. Collectively, this trend reflects a scaling law‐driven paradigm in modern artificial intelligence, where increasing model size is closely linked to improved generalization, emergent capabilities, and enhanced cross‐domain adaptability.

Among these technologies, LLMs have attracted particular attention because of their powerful capabilities in natural language understanding, knowledge reasoning, and multimodal information integration [26, 27, 28, 29, 30, 31, 32, 33, 34] (Figure 1). By leveraging large‐scale corpora of biomedical literature, electronic health records (EHRs), radiology reports, pathology reports, and clinical guidelines, LLMs can support cancer screening, diagnostic reasoning, prognosis prediction, treatment planning, patient education, and clinical trial matching [35, 36, 37, 38, 39, 40, 41, 42, 43, 44]. At the same time, other generative AI approaches are becoming increasingly important in oncology. GANs and diffusion models can generate realistic radiological and pathological images to address data scarcity and class imbalance, while multimodal foundation models enable joint learning across imaging, pathology, genomics, and clinical text [45, 46, 47, 48]. Together, these technologies are reshaping the development of intelligent oncology systems by enabling data generation, knowledge synthesis, and cross‐modal clinical reasoning (Table 1).

**TABLE 1:** Overview of domain‐specific LLMs for medicine.

Model name	Training data	Model architecture	Multimodal support	Applications	Ops/resources	Repro pack	Year	References
Med‐PaLM	MultiMedQA	PaLM(540B)	No	QA	Proprietary/closed‐source	Proprietary/closed‐source	2023	[26]
Med‐PaLM 2	MultiMedQA	PaLM 2	No	QA	Proprietary/closed‐source	Proprietary/closed‐source	2025	[23]
AMIE	Simulated clinical dialogues, medical QA datasets, real‐world OSCE transcripts, CPC reports from NEJM	PaLM 2	No	Differential diagnosis, history‐taking, dialogue simulation, medical QA, and decision support	Proprietary/closed‐source	Proprietary/closed‐source	2025	[19]
MedFound	MedCorpus	BLOOM (176B)	No	Disease diagnosis	176B‐parameter medical LLM trained on large‐scaleMedCorpus; compute infrastructure not reported	GitHub repository available (https://github.com/medfound/medfound); evaluation datasets released via the repository; model card and Docker environment not reported	2025	[51]
MEDLM‐176B	MedCorpus	176‐Billion‐Parameter LLM	No	Diagnostic reasoning, EHR summarization and interpretation	Proprietary/closed‐source	Proprietary/closed‐source	2025	[24]
Med‐MLLM	Visual data; textual data; multimodal data	Image encoder: ResNet‐50; text encoder/decoder: transformer‐based (BERT variant with radiology‐specific vocabulary)	Yes	Automatically generates medical reports, classifies diseases, predicts patient survival outcomes	Proprietary/closed‐source	Proprietary/closed‐source	2023	[36]
MetaGP	EHRs; academic articles; medical books; multimodal data	Qwen‐1.5 (32B)	Yes	Rare disease diagnosis, emergency condition identification, medical report generation, clinical decision support	Pretraining on 120 NVIDIA A100 (80 GB) GPUs; instruction tuning on 48 A100 GPUs; multimodal foundation model integrating EHR and imaging	Uses multiple public benchmarks (PubMedQA, MedQA, MedMCQA); code/data availability partially released or planned	2025	[34]
SkinGPT‐4	Skin disease images, image‐concept pairs, image‐diagnosis pairs	Llama‐2‐13b	Yes	Interactive dermatological diagnosis, automated report generation, preliminary diagnosis	Training on 8 NVIDIA A100 (80 GB) GPUs (∼24 h); deployment requires GPU ≥30 GB memory (e.g., V100); CPU possible but slower	GitHub repository available (SkinGPT‐4); datasets include SKINCON and DermNet; environment specification not reported	2024	[52]

**FIGURE 2:**
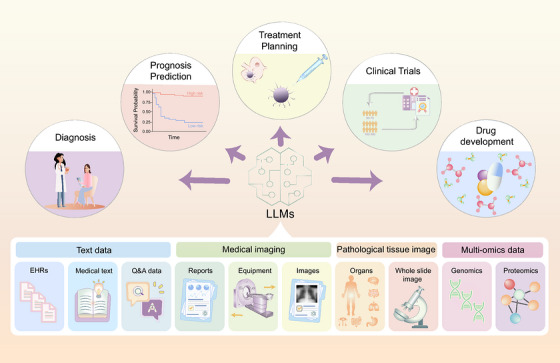
Applications of LLMs in cancer diagnosis and treatment. This figure illustrates the central role of LLMs in integrating multimodal medical data across the oncology workflow. By aligning clinical text, imaging, pathology, and omics data into unified semantic representations, LLMs improve diagnostic consistency and enable personalized treatment strategies. Importantly, these models facilitate a shift from reactive treatment toward predictive and preventive oncology. Such capabilities may enhance clinical efficiency, reduce variability, and support data‐driven precision medicine.

Given the rapid expansion of generative AI applications in oncology, a comprehensive review of both LLMs and other generative AI technologies is timely and necessary [49, 50]. In this review, we first summarize the major categories of generative AI relevant to cancer research and clinical practice, including LLMs, GANs, diffusion models, and multimodal foundation models (Box 1). We then discuss their applications in cancer diagnosis, prognosis prediction, treatment planning, patient management, and clinical trials (Figure 2). Finally, we examine current challenges related to interpretability, reliability, privacy protection, regulatory governance, and clinical implementation, and highlight future directions for developing trustworthy and clinically deployable generative AI systems in precision oncology.

**BOX 1 Emerging generative AI technologies and terminology**

To ensure clarity, key terms used in this review are defined below:

- **Multimodal AI**: Artificial intelligence systems capable of integrating and jointly reasoning over heterogeneous data types—such as text, imaging, genomic, and structured data—thereby enabling richer, context‐aware decision‐making in complex clinical environments.
- **Generative AI**: A class of models designed to synthesize new content, including text, images, or audio. Generative AI is widely employed in clinical documentation, virtual simulation, and the creation of synthetic datasets for model training and validation.
- **GANs**: A generative learning framework consisting of two competing neural networks: a generator that synthesizes realistic data samples and a discriminator that distinguishes generated data from real data. Through adversarial training, GANs can produce high‐fidelity synthetic medical images, augment limited datasets, reduce class imbalance, and facilitate privacy‐preserving data sharing in oncology research and clinical applications.
- **Diffusion Models**: A class of generative models that learn to gradually reverse a noise‐corruption process to generate high‐quality synthetic data. Diffusion models have demonstrated remarkable performance in medical image synthesis, reconstruction, super‐resolution, and modality translation.
- **Multimodal Foundation Models**: Large‐scale pretrained models designed to jointly learn representations from multiple data modalities, including text, medical images, pathology slides, genomic profiles, and EHRs. By aligning heterogeneous information within a shared representation space, multimodal foundation models enable cross‐modal understanding, reasoning, and generation, supporting tasks such as integrated diagnosis, prognosis prediction, treatment recommendation, and clinical decision support.
- **Digital Twin**: A computational construct representing a dynamic, virtualized replica of an individual patient. Digital twins integrate longitudinal clinical data to support personalized treatment planning, real‐time monitoring, and outcome simulation.
- **Multihead Self‐Attention**: A core component of transformer architectures that enables the model to attend to multiple positions in the input sequence simultaneously across different representation subspaces (heads), capturing diverse contextual relationships.
- **Vision Transformers (ViTs)**: Transformer‐based architectures adapted for computer vision tasks. Images are partitioned into fixed‐size patches, embedded as token sequences, and processed via self‐attention to model global dependencies, enabling applications in classification, segmentation, and detection.

- **Knowledge Distillation**: A model compression strategy wherein a smaller student model is trained to approximate the behavior of a larger, more complex teacher model. By mimicking softened output distributions, the student absorbs not only final predictions but also intermediate knowledge representations.
- **Instruction Tuning**: A fine‐tuning paradigm where pretrained language models are adapted using datasets of input–output pairs framed as natural language instructions. This technique enhances the model's ability to follow human prompts and improves generalization across a broad spectrum of tasks.
- **Fusion Embedding**: A representation learning strategy that projects features from multiple modalities (e.g., text, image, structured data) into a shared embedding space, facilitating unified reasoning. It underpins many multimodal applications such as medical report generation and cross‐modal retrieval.
- **Multiturn Dialogue Modeling**: A conversational modeling technique that captures contextual dependencies across multiple dialogue exchanges. It enables dialogue systems to maintain coherence, track user intent, and generate contextually relevant responses, essential for intelligent agents in clinical consultations and patient triage.
- **Image‐Text Contrastive Learning**: A self‐supervised paradigm in which models learn to align visual and textual representations by pulling matched pairs closer in embedding space while pushing mismatched pairs apart. This technique is foundational to models such as CLIP and ALIGN.

## 2 Generative AI and LLMs in Cancer Diagnosis

Cancer diagnosis increasingly relies on integrating multimodal data, including medical imaging, EHRs, genomic profiles, and histopathological images. However, the high heterogeneity, unstructured nature, data incompleteness, and inconsistent formats of such sources significantly complicate data processing and clinical interpretation [53, 54, 55, 56]. Recent advances in generative AI have provided powerful solutions to these challenges. Encompassing LLMs, GANs, diffusion models, and multimodal foundation models, generative AI can not only generate realistic biomedical data but also enable multimodal representation learning and cross‐modal reasoning [16, 22, 51, 57, 58]. Among these approaches, LLMs have demonstrated remarkable capabilities in processing clinical narratives, imaging reports, genomic information, and laboratory data, facilitating automated report generation and clinical decision support [51, 57, 58]. Through cross‐modal attention mechanisms, transformer‐based models align textual descriptions, image features, and molecular signals within a shared embedding space, thereby enhancing diagnostic reasoning and multimodal information fusion [22, 59, 60]. Models such as Flamingo and GPT‐4o further enable semantic integration across text and imaging modalities through learnable modality‐adaptation modules [61, 62]. Meanwhile, GANs and diffusion models can generate realistic radiological and pathological images to alleviate data scarcity and class imbalance, whereas multimodal foundation models support unified learning from imaging, pathology, genomics, and clinical records [63, 64, 65, 66]. For example, HistoXGAN employs a GAN to reconstruct histopathology images from latent feature representations, facilitating tumor subtype identification, molecular feature interpretation, diagnostic decision support, and virtual biopsy generation [63]. Collectively, these generative AI technologies are reshaping cancer diagnosis by enabling more efficient data integration, knowledge extraction, and personalized clinical decision‐making.

Medical imaging modalities such as X‐rays, CT, and MRI are pivotal in cancer diagnosis. However, traditional image interpretation depends heavily on radiologists’ expertise, introducing variability and subjectivity [67]. Generative AI offer innovative solutions for automating and standardizing radiology reporting by integrating visual and textual features. This accelerates report generation, reduces human error, and improves diagnostic precision [62, 68, 69]. For example, Flamingo‐CXR employs Swin Transformer‐based visual encoders to extract high‐dimensional features, which are then jointly trained with text generation modules to produce consistent and accurate radiological descriptions [62]. Additionally, LLMs can support staging decisions and assess tumor resectability, further advancing intelligent cancer diagnostics [64]. One persistent challenge is the limited availability of imaging data for rare cancer types, which restricts model generalizability in real‐world clinical settings. Generative AI has emerged as a transformative solution by synthesizing imaging data for underrepresented cancers. In addition to data augmentation, diffusion models have demonstrated remarkable capabilities in medical image synthesis, reconstruction, super‐resolution, and modality translation. These models can generate realistic CT, MRI, PET, and mammography images while preserving clinically relevant anatomical structures [70, 71]. Such synthetic data improve the performance of downstream tasks, including tumor detection, segmentation, classification, and biomarker prediction, particularly in rare cancers and low‐resource clinical settings [71, 72]. Furthermore, GAN‐based approaches have been widely applied to low‐dose image enhancement and cross‐modality image synthesis, improving image quality and diagnostic consistency while reducing acquisition costs and radiation exposure [73].

Histopathology, often regarded as the diagnostic gold standard in oncology, emphasizes microscopic structural analysis and complements the macroscopic insights from imaging. Due to the high resolution of pathology slides, multiscale attention mechanisms are essential for capturing both local morphology and global architecture. Generative AI can effectively extract critical features from pathological images and integrate them with clinical history to generate structured reports, thereby providing efficient support for pathological diagnosis [74]. Weakly supervised and multiple‐instance learning approaches are widely adopted to circumvent the need for expensive pixel‐level annotations. For example, the Prov‐GigaPath model, built on a visual transformer architecture, can predict cancer‐associated mutations, perform subtyping, and generate structured reports to guide precision medicine [75]. The Virchow model combines multiscale convolution and visual transformer techniques to identify rare cancers with high sensitivity [76]. The CHIEF model supports prognosis prediction by analyzing microstructural features and identifying tumor origin and key genomic markers [77] (Table 2). GANs and diffusion models can generate realistic histopathological patches, perform stain normalization, and enable virtual staining or virtual immunohistochemistry, reducing dependence on costly laboratory procedures. Synthetic pathology images have been shown to improve model performance for tumor classification, biomarker prediction, and rare cancer recognition [78, 79].

**TABLE 2:** Overview of domain‐specific foundation models for medicine.

Model name	Training data	Model architecture	Applications	Year	References
CHIEF	60,530 WSIs from 19 anatomical sites; 15 million tiles; validated on 19,491 slides	Self‐supervised tile‐level encoder; weakly supervised WSI‐level model with CLIP‐style contrastive embedding	Cancer detection, tumor origin prediction, mutation prediction (e.g., TP53, GTF2I), MSI/IDH status, survival prediction	2024	[77]
MUSK	50M unpaired pathology images from 11,577 patients (33 cancer types); 1B pathology‐related text tokens; 1M image–text pairs from QUILT‐1M	BEiT3‐inspired transformer; shared self‐attention + expert branches for vision and language; two‐phase pretraining: masked modeling + contrastive alignment	Cancer detection, biomarker prediction (e.g., HER2, IDH), prognosis, relapse risk, immunotherapy response (lung, gastric), VQA, classification, retrieval	2025	[58]
Prov‐GigaPath	1.38B image tiles (256 × 256) from 171,189 WSIs across 30,000+ patients and 31 tissue types	DINOv2‐based ViT tile encoder + LongNet slide encoder with masked autoencoding	Cancer subtyping (9 types), mutation prediction (e.g., EGFR, KRAS), tumor burden, vision–language retrieval, zero‐shot inference	2024	[75]
Virchow	1.5 million H&E whole‐slide images	ViT‐H (632M parameters), trained with DINOv2; tile embeddings aggregated via simple aggregator networks	Pan‐cancer detection, rare cancer detection, biomarker prediction (e.g., EGFR, HER2, BRAF), subtype classification, clinical‐grade AI comparisons	2024	[76]
FastGlioma	4 million stimulated Raman histology images	Patch tokenizer + vision transformer; fine‐tuned with ordinal metric learning	Intraoperative detection of glioma infiltration, tumor grading, margin assessment, glioma subtype stratification; zero‐shot generalization to other brain tumors	2025	[74]
MINIM	Paired image–text data across 6 modalities (OCT, fundus, chest X‐ray, chest CT, brain MRI, breast MRI)	Latent stable diffusion model with self‐improvement via RLHF and transfer learning	Synthetic image generation, diagnosis, report generation, self‐supervised learning, mutation prediction (EGFR, HER2), survival analysis	2025	[72]
M3FM	163,725 chest CT scans; 49 types of clinical data; 17 clinical tasks	CT vision transformer, text transformer, task encoder, multimodal predictors	Lung nodule detection and characterization, lung cancer risk prediction (1–6 years), cardiovascular disease (CVD) diagnosis and mortality prediction, COVID‐19 detection, lung‐RADS classification, various chest abnormality diagnoses	2025	[84]
RETFound‐DE	1.26M synthetic retinal images (via stable diffusion) + 150,786 real CFP images (16.7% of RETFound data)	Vision transformer, pretrained with MAE	Diabetic retinopathy grading, glaucoma diagnosis, AMD Grading, multidisease classification; also validated on chest X‐ray for tuberculosis	2025	[88]
M^3^FM	MIMIC‐CXR, COVID‐19 CT/CXR datasets, machine‐ and human‐translated chinese reports; trained on CXR–EN, CT–EN, and EN–ZH pairs	MultiMedCLIP; MultiMedLM; transformer‐based encoders and decoders	Zero‐/few‐/full‐shot clinical report generation and disease classification across domains and languages, especially for rare and new diseases	2025	[89]
MedIM	MIMIC‐CXR V2 (377,110 chest X‐rays with reports); evaluation on NIH, CheXpert, RSNA, SIIM‐ACR, MS‐CXR datasets	ViT‐B/16 (image encoder) + BERT (text encoder + ITM decoder); multiscale contrastive learning	Disease classification (fine‐tuning, zero‐shot), pneumothorax segmentation, zero‐shot phrase grounding	2024	[90]
BiomedGPT	592,567 images, 183M Text sentences, 271,804 image–text pairs, 46,408 object–label pairs from 14 public datasets	Encoder–decoder transformer; versions: small (33M), medium (93M), base (182M); unified vocabulary (text + image + location)	VQA, report generation, summarization, classification, lesion detection, treatment recommendation, trial matching, mortality prediction	2024	[91]
PLIP	208,414 pathology image–text pairs from Twitter and LAION (OpenPath Dataset)	CLIP‐based contrastive learning: ViT‐B/32 (image encoder) + transformer (text encoder)	Zero‐shot classification, linear probing, text‐to‐image and image‐to‐image retrieval in pathology	2023	[53]

*Abbreviations*: whole slide images (WSIs); optical coherence tomography (OCT); reinforcement learning from human feedback (RLHF); age‐related macular degeneration (AMD).

Generative AI also holds great promise for early cancer screening. In breast cancer, for example, AI‐assisted screening using imaging data has demonstrated clinical utility [80, 81, 82]. The RadioLOGIC system utilizes natural language processing to extract “repomics” features, defined as semantic radiomics descriptors, from unstructured reports for predicting BI‐RADS scores and pathological outcomes, thereby delivering reliable clinical decision support [83]. This approach is now being extended to other cancer types. Self‐supervised and transfer learning strategies are increasingly employed to address label scarcity in early screening scenarios. The M3FM model, for instance, uses image–text contrastive pretraining to enable cross‐cancer knowledge transfer and enhance rare subtype recognition [84]. GPT‐4o has also shown strong performance in longitudinal CT analysis for pulmonary nodules, accurately assessing changes in malignancy probability, volume, and morphology—thereby supporting early lung cancer detection and surveillance [61]. Despite the promising advances, LLMs’ performance remains highly dependent on the quality and diversity of training data [85]. Biases related to race, geography, or cultural context can lead to uneven model performance across populations [13]. Uncertainty quantification remains a key challenge in clinical deployment, with methods such as Monte Carlo dropout and ensemble models being explored to improve robustness for rare diseases [86]. Moreover, current LLMs still struggle with atypical or edge cases due to the underrepresentation of such examples in training data, leading to compromised prediction reliability [87]. Overreliance on AI tools may also diminish clinicians’ critical evaluation of outputs, introducing additional risk. To ensure the safe and effective integration of LLMs into cancer diagnostics, future efforts must focus on mitigating data bias, improving model generalizability and robustness, and conducting thorough validation in real‐world clinical environments.

## 3 Prognosis Prediction Using Generative AI and LLMs

Accurate prognosis prediction is critical for guiding personalized cancer treatment and long‐term disease management. By learning complex patterns from multimodal biomedical data, generative AI can generate robust representations, impute missing information, model disease trajectories, and uncover latent associations among clinical, imaging, pathological, and molecular features. HistoPlexer is a conditional GAN that predicts spatially resolved multiplexed protein expression directly from routine H&E images, enabling accurate tumour microenvironment characterization and improving prognostic and immune subtype prediction [92]. LLMs have emerged as particularly powerful tools for prognosis prediction. Predominantly based on Transformer architectures, LLMs leverage multihead self‐attention mechanisms to model long‐range dependencies within unstructured clinical notes and longitudinal time‐series data, making them well suited for complex cancer trajectory prediction tasks [93]. Unlike traditional recurrent neural networks and long short‐term memory networks, transformers demonstrate superior capabilities in capturing long‐term dependencies, enabling parallel training, and preserving contextual semantics [94]. LLMs and foundation models can effectively integrate unstructured EHR data with multiomics modalities (e.g., genomics and proteomics) to enable dynamic disease progression monitoring. This capability facilitates more accurate survival time prediction, recurrence risk, and other critical clinical outcomes, thereby providing robust decision support for clinicians [95, 96]. For instance, using NLP techniques to extract free‐text clinical data from the MSK‐CHORD multimodal dataset, researchers developed prognostic models that significantly improved survival predictions. Studies have shown that AI models integrating clinical and genomic data outperform those relying solely on genomic information [54]. The CHIEF model, constructed from histopathological images, not only supports cancer diagnosis but also predicts survival outcomes, tumor molecular features, and treatment response [77]. By leveraging cross‐modal attention mechanisms, these models align features from pathology images, molecular profiles, and clinical texts within a shared embedding space, enabling coordinated multimodal learning. The MUSK foundation vision–language model demonstrated strong performance across 16 cancer types by fusing pathology images and clinical notes, effectively stratifying patients by risk, and capturing survival heterogeneity [58]. MUSK employs a joint attention layer to semantically map intratumoral heterogeneity captured in pathology slides to relevant clinical descriptors, thereby enhancing the accuracy and interpretability of survival predictions [58].

EHRs are stored in free‐text formats, which are challenging to process computationally and remain underutilized in AI modeling. Manual extraction of key information by clinicians is labor intensive and time consuming. NLP offers efficient solutions for processing unstructured EHR data, facilitating key information retrieval and clinical report interpretation [97]. For example, NLP models can extract cancer progression and treatment response from radiology reports of lung cancer patients, achieving high accuracy and significant correlation with overall survival outcomes [98]. Notably, NLP models trained on initial oncology visit notes achieved exceptional discrimination (AUC 0.928) in predicting survival at 6, 36, and 60 months [99]. In recent years, self‐supervised learning has been increasingly employed to pretrain EHRs and omics data, reducing reliance on manual annotations and improving model robustness in unseen scenarios. Pretraining tasks such as time masking, sentence ordering, and contrastive learning allow models to acquire generalizable semantic representations from large volumes of unlabeled clinical data. Knowledge distillation frameworks, particularly the teacher–student paradigm, have been proposed to improve model transferability and enhance privacy protection. These approaches extract prognostic knowledge from multicenter radiology reports and clinical notes, achieving strong performance (AUROC > 0.90) on external validation cohorts while mitigating direct exposure of sensitive data [100]. Teacher–student distillation compresses complex multicenter models into lightweight student networks, addressing interinstitutional heterogeneity and facilitating privacy‐preserving deployment. Meanwhile, the PRISSMM model, built using NLP, enables structured extraction of cancer outcomes, such as survival, progression, treatment response, and metastatic sites, from radiology reports and oncologist notes [101]. Multimodal AI models integrating omics data (e.g., genomics, proteomics, transcriptomics) with EHRs demonstrate significant potential in cancer prognosis modeling by capturing molecular mechanisms and clinical trajectories [102, 103].

Despite these advances, data privacy and model interpretability remain significant challenges [96, 104]. EHRs and omics datasets contain sensitive patient information, and centralized training or data sharing may pose privacy risks [105]. Although privacy‐preserving technologies such as federated learning (FL) and differential privacy have been explored, practical deployment in clinical environments is hindered by high communication overhead, hardware heterogeneity, and limited computational resources [105, 106, 107]. In FL, models are trained locally at individual institutions, with only model updates being shared, thus avoiding centralized data transfer. Techniques such as FedAvg and FedProx enable model aggregation in decentralized environments. However, deployment challenges include communication delays, non‐IID data distributions, and unequal device capabilities. Recent advancements, such as clustered FL and personalized FL (e.g., pFedMe), aim to enhance model stability and adaptability across diverse clinical settings. Interpretability remains another barrier to clinical adoption. While LLMs often achieve high predictive performance, their “black‐box” nature can limit trust and usability among healthcare professionals [108]. To address these challenges, it is essential to optimize privacy‐preserving techniques further while developing more transparent and interpretable models, including attention‐based architectures and integrated visualization tools, to support clinical decision‐making better [91]. Currently, widely adopted interpretability methods include attention heatmaps, SHAP (Shapley Additive exPlanations) values, and integrated visualization frameworks such as Grad‐CAM and LIME. These approaches help elucidate the model's focus on critical input variables, thereby assisting clinicians in understanding the rationale behind predictions [95, 109]. In addition, multimodal interpretability models providing longitudinal decision pathways offer more trustworthy support for formulating personalized treatment strategies [110, 111].

## 4 Generative AI and LLMs in Cancer Treatment Planning

Cancer treatment continues to confront two persistent challenges, specifically the unequal distribution of medical resources and the absence of standardized decision‐making protocols. Breast cancer serves as a representative example, with marked disparities observed between Chinese and American patient populations regarding diagnostic age, disease stage at presentation, and therapeutic approaches. Epidemiological data reveal that Chinese patients typically receive diagnoses at younger median ages, yet demonstrate higher frequencies of advanced‐stage disease at initial presentation. While targeted therapeutic agents were introduced later in the Chinese healthcare system, incorporating trastuzumab and pertuzumab into national medical insurance coverage has significantly improved treatment accessibility and protocol standardization. Notably, among HER2‐positive patients, utilization rates of targeted therapies have demonstrated a dramatic increase from 8.6 to 88.9% [112]. Nevertheless, deviations from established treatment guidelines persist, particularly in primary healthcare institutions. By integrating heterogeneous data from clinical records, medical imaging, pathology, molecular profiling, and treatment outcomes, generative AI can support treatment‐response prediction, biomarker discovery, virtual patient simulation, and personalized therapeutic recommendation. Among these technologies, LLMs have emerged as particularly valuable tools for clinical decision support. LLMs leverage advanced NLP techniques to integrate multimodal inputs, encompassing medical literature, clinical guidelines, and patient‐specific clinical data, thereby achieving comprehensive medical knowledge understanding and enhanced clinical decision support [113]. Fine‐tuning strategies such as instruction tuning and reinforcement learning with human feedback have substantially improved model generalizability in complex medical contexts [91]. Moreover, multitask learning frameworks allow LLMs to perform tasks such as tumor subtyping, treatment recommendation, and drug response prediction, thereby improving clinical efficiency and accuracy [102]. By incorporating patient‐specific features, LLMs can generate personalized and precise treatment suggestions to optimize physician decision‐making [91].

While featuring distinct architectural designs, LLMs systems, including DeepSeek, ChatGPT, Gemini 2.0, and Grok 3, consistently demonstrate robust logical reasoning and multimodal data processing capabilities. Using knowledge distillation, LLMs can be compressed into lightweight versions suitable for edge deployment in hospital settings. Combined with FL, these models can be deployed locally while preserving patient privacy and ensuring real‐time responsiveness. The teacher–student framework retains high performance while significantly reducing parameter size and inference latency, making it feasible for edge devices [114]. FL frameworks further enhance privacy through DP‐FedAvg and secure aggregation, which encrypt model parameters and eliminate the need to transmit raw medical data across institutions [115]. These technological integrations facilitate a shift from traditional empirical medicine to intelligent, data‐driven precision medicine. Generative AI enable standardized decision‐making in under‐resourced settings via real‐time knowledge updates while assisting clinicians in overcoming cognitive limitations inherent to experience‐based practice. In complex scenarios such as molecular subtyping and treatment sequencing, generative AI enable dynamic optimization of treatment plans, establishing a human‐AI collaborative framework for precision oncology. Generative AI has demonstrated high utility across various cancer care domains, including diagnosis, therapeutic decision‐making, and rehabilitation, by providing personalized, context‐aware clinical recommendations [116, 117]. By integrating multimodal data, including molecular subtypes, genomic mutation profiles, clinical histories, and treatment responses, generative AI can optimize therapeutic strategies, including chemotherapy, immunotherapy, and targeted therapy [118, 119]. Fusion embedding and cross‐attention mechanisms are commonly used to harmonize these diverse data types into coherent representations [120]. Inspired by architectures such as VisualBERT and CLIP, LLMs can align and jointly encode medical images with textual descriptions to enhance understanding of individual patient characteristics [121, 122]. For incorporating molecular data such as WES/WGS, GNNs are employed to model mutation interaction networks, thereby improving the accuracy of targeted therapy recommendations [123]. These models can predict drug efficacy and adverse effects, adjust chemotherapy dosing and scheduling, and identify immunotherapy candidates based on tumor mutational burden and immune microenvironment features. Additionally, they can detect driver mutations to guide dynamic adjustments in targeted therapies, ultimately advancing personalized cancer treatment [124, 125, 126, 127].

Beyond clinical decision support, generative AI hold broad potential in cancer patient education, treatment guidance, and survivorship care. As intelligent consultation tools, LLMs can rapidly provide patients with key information regarding their condition, treatment options, and daily care management [128]. Multiturn dialogue modeling, dialogue state tracking, and policy optimization enable more coherent and context‐aware interactions during patient follow‐ups. Reinforcement learning methods such as proximal policy optimization and expert feedback can further enhance medical accuracy and linguistic naturalness of responses [129, 130]. Based on the patient's disease profile and treatment stage, LLMs can generate personalized educational content, presented in an accessible and empathetic manner, to improve health literacy and treatment adherence [131]. LLMs can increase patient engagement and reduce psychological distress by providing easy‐to‐understand cancer education and emotional support for sensitive topics, ultimately improving overall treatment outcomes [96]. While LLMs typically exhibit high accuracy in addressing common cancer‐related queries, their ability to provide evidence‐based therapeutic recommendations remains limited [132, 133]. Studies have found that although some treatment suggestions generated by LLMs align with clinical guidelines, a considerable proportion contain hallucinated or erroneous content. These inaccuracies, often embedded within otherwise credible outputs, can be difficult for experts to detect, posing a significant risk of misinformation. Therefore, in cancer patient education and management, LLMs should be considered supplementary tools rather than primary sources of medical guidance [134].

Ensuring the accuracy and reliability of LLMs’ outputs is thus a critical concern. This not only influences patient trust and engagement but also impacts treatment outcomes. Future research should focus on improving the fidelity and transparency of LLMs. On the one hand, constructing larger, more comprehensive medical knowledge bases and employing rigorous training protocols are essential to reduce bias and misinformation [135]. On the other hand, integrating explainable AI techniques—such as attention visualization and model rationale tracing—can enhance clinicians’ confidence in model outputs [136, 137, 138]. Finally, aligning LLMs development with real‐world clinical workflows and incorporating continuous feedback from medical professionals will be key to improving the utility of LLMs in cancer education, treatment planning, and patient‐centered care.

## 5 Applications of Generative AI and LLMs in Specific Cancer Types

### 5.1 Breast Cancer

AI has demonstrated significant promise in breast cancer screening and precision care. Notably, the Mirai model leverages deep learning to perform multitimepoint risk predictions directly from mammographic images without relying on complete clinical information. It exhibits strong cross‐device and cross‐population generalizability and significantly outperforms traditional risk models, enabling early identification of high‐risk individuals [139]. In a multicenter, prospective real‐world study, the PRAIM project further validated the clinical feasibility and effectiveness of AI‐assisted screening. The study showed that an AI‐supported double‐reading mechanism outperformed traditional human double‐reading in cancer detection rates. Importantly, this improvement was achieved without a significant increase in recall rates, thereby enhancing the positive predictive value and improving screening efficiency without increasing the risk of overdiagnosis [140]. Despite the promising results of Mirai and PRAIM, the broader implementation of AI models in different healthcare systems still faces challenges related to data privacy, model bias, and ethical governance. These challenges are particularly pronounced in low‐resource settings where data quality and availability are limited, raising concerns about model generalizability. With advances in cross‐institutional FL, future AI‐based breast cancer screening systems may adopt decentralized collaborative training paradigms. This approach would enable data privacy preservation while breaking institutional data silos, ultimately enhancing model adaptability and equity across diverse clinical environments [141].

In recent years, generative AI have shown growing capabilities in integrating multiple medical data sources to facilitate knowledge fusion and clinical reasoning. This advancement significantly enhances their potential applications in breast cancer diagnosis and therapeutic decision support [44, 49]. For instance, LLMs can automatically extract clinically relevant features from unstructured radiology reports to accurately predict BI‐RADS classifications and assist in interpreting pathological outcomes, offering intelligent decision support for radiologists [142]. With their capacity for multimodal data integration, LLMs can jointly process clinical text and imaging data to support the automatic delineation of radiotherapy target regions, accurately determine tumor staging (T/N) and laterality, and recommend surgical procedures. This reflects their growing ability to understand and apply complex clinical semantic information [120]. Despite these advances, current LLMs still face limitations such as ambiguity in semantic interpretation, outdated medical knowledge, and insufficient model interpretability. For example, BI‐RADS classification heavily relies on a nuanced contextual understanding of radiological language, and existing LLMs remain prone to “hallucinatory” reasoning. As multimodal models like GPT‐4V evolve, future LLMs are expected to integrate deeply with domain‐specific knowledge graphs and ontologies like RadLex. This enables traceable and interpretable reasoning paths that are more suitable for complex radiology and pathology tasks [143]. Notably, in breast cancer prevention and screening, LLMs have shown significant potential in health education, clinical communication, and patient‐centered decision support by providing accurate and personalized responses to common patient queries [144]. In addition, the MINIM model has enhanced HER2 mutation prediction in breast cancer by generating high‐quality synthetic medical images. By leveraging joint training on photos and textual descriptions, MINIM produces synthetic images that are visually accurate and semantically aligned with key clinical report features. When combined with real‐world imaging data, this approach significantly improved HER2 mutation classification accuracy, increasing from 79.2 to 94.0%. This enhanced precision in identifying HER2‐positive patients enables more optimized targeted therapy decision‐making [72]. AI models in breast cancer care are expected to evolve toward human–AI coexplanation and self‐supervised knowledge transfer paradigms. These models will align image data, pathology narratives, and genomic information at a deeper semantic level, forming verifiable diagnostic chains that enhance interpretability and clinical reliability.

Histopathology plays a pivotal role in the precision diagnosis and treatment of breast cancer. Developing high‐quality pathology foundation models tailored to specific diseases is a critical step toward improving the performance of histological image classification and molecular status prediction. Research indicates foundation models with multiple instance learning can effectively grade breast cancer histology (e.g., distinguishing high versus low risk) and predict three critical biomarkers, namely, ER, PR, and HER2 [145]. Meanwhile, several pan‐cancer pathology foundation models have demonstrated excellent performance in breast cancer applications. These models achieve strong results in subtype classification and prognostic stratification, effectively identifying high‐risk patients while supporting personalized treatment strategy development [102, 146].

GPT‐4V has exhibited remarkable performance in breast cancer diagnostic applications. The model demonstrates dual capabilities in detecting lymph node metastases from histopathological images and generating logical, interpretable diagnostic reasoning. These capabilities underscore its advantages in clinical reasoning, knowledge transfer, and model explainability, particularly for complex diagnostic situations encountered in real‐world clinical practice where labeled data are scarce and model fine‐tuning is not feasible [94]. Notably, ConcepPath achieves deep integration of LLMs with vision–language models. ConcepPath enables semantic alignment with histopathological whole‐slide images by incorporating a multiple‐instance learning strategy. In the HER2 scoring task, ConcepPath utilizes GPT‐4 to distill and summarize domain‐specific knowledge about HER2 status from biomedical literature and further integrates complementary features learned autonomously from training data. This allows for hierarchical aggregation of image‐level features and precise HER2 status prediction. On the TCGA‐BRCA dataset, ConcepPath demonstrated high accuracy and strong interpretability in HER2 status classification, offering robust support for molecular subtyping and personalized treatment planning in breast cancer care [147].

In addition, the Protein Importance Calculator, a protein‐focused LLM, has developed a hierarchical framework for predicting protein essentiality across human, cellular, and murine systems. Using breast cancer as a representative case, the study validated the practical utility of its proposed Protein Essential Score in both biomarker discovery and survival prediction. This work presents an innovative, LLM‐driven strategy for precision oncology in breast cancer care. The model exemplifies a novel pathway toward personalized breast cancer treatment, showcasing how the deep integration of LLMs with domain‐specific biological knowledge can drive transformative changes in diagnostic and therapeutic paradigms [148].

### 5.2 Lung Cancer

Generative AI have demonstrated significant potential in assisting lung cancer screening. Prompt‐based LLMs can process patient inquiry data and automatically generate structured medical records, improving screening efficiency and clinical decision‐making precision. Moreover, integrating speech recognition technology into the workflow further increases the system's practical utility, particularly in reducing the time required for initial screening and improving overall clinical productivity [149]. The multimodal and multitask medical foundation model M3FM integrates low‐dose CT imaging, demographic information, and EHRs to effectively process high‐dimensional imaging data while incorporating patients’ clinical context. This model excels in multiple tasks, including pulmonary nodule detection, lung cancer risk assessment, and cardiovascular disease prediction, significantly enhancing the accuracy and efficiency of lung cancer screening [84]. With the advancement of integrative analysis across biospecimens, imaging, and clinical text, future AI‐driven lung cancer screening systems may enable a fully integrated early detection paradigm ranging from auscultatory sounds to blood‐based analysis, offering noninvasive, rapid, and cost‐effective advantages. In addition, a liquid biopsy test powered by deep generative AI analyzes serum‐derived orphan noncoding RNAs to detect NSCLC. The Orion model demonstrated a sensitivity of 94% in early‐stage lung cancer detection, including 90% sensitivity in stage I, significantly outperforming traditional support vector machines (SVMs). Orion also maintained high sensitivity and strong generalizability across different tumor stages and sizes, highlighting its promising clinical applicability [150].

LLMs are increasingly pivotal in lung cancer diagnosis and treatment by leveraging EHRs. ChatGPT has demonstrated the ability to extract structured information from free‐text pathology reports of lung cancer, accurately identifying pathological classifications, tumor staging, and molecular features. This highlights its potential to rapidly and efficiently retrieve key clinical data without requiring extensive manual annotation or additional model training, offering substantial value for clinical decision support and research applications [151]. GPT‐4 further extends this capability by extracting and annotating tumor phenotypes from unstructured CT radiology reports. It can identify lesion measurements, detect metastatic disease, and categorize tumor progression, underscoring its potential to enhance the efficiency and scalability of radiological data mining in clinical practice [152]. In addition, GPT‐4o has been validated for assessing malignancy risk, size progression, and phenotypic features of pulmonary nodules directly from CT images. GPT‐4o demonstrated superior malignancy risk prediction accuracy compared with radiologist assessments and pathological diagnoses, with follow‐up imaging further enhancing performance, as reflected by a significant increase in AUC. The model's nodule size estimation and feature detection performance are closely aligned with expert radiologists, providing high‐quality radiological evidence for lung cancer screening and longitudinal monitoring [61]. With the advancement of multilingual pretraining frameworks (e.g., XLM‐R, mGPT) and domain‐specific fine‐tuning for healthcare, future LLMs are expected to offer enhanced language generalizability, cultural adaptability, and intelligent conversational capabilities. These developments promise to promote equitable and cross‐cultural accessibility in lung cancer care.

Generative AI can effectively integrate multimodal data such as EHRs, chest CT scans, and pathology reports to extract lung cancer biomarkers automatically, enhancing clinical decision support [88, 89, 90]. The MUSK vision–language foundation model predicts immunotherapy response and progression‐free survival in NSCLC patients by combining pathological images with clinical reports. Compared with conventional biomarkers such as PD‐L1 expression, MUSK achieved significantly higher predictive performance, with an AUC of 0.768 and a C‐index of 0.705. It also outperformed other multimodal models based solely on pathology images or text reports, highlighting its substantial potential in precision immuno‐oncology [90]. In addition, self‐supervised deep learning models trained on large‐scale CT datasets encompassing 11,467 lesions have proven effective in malignancy prediction of pulmonary nodules, prognosis prediction in NSCLC, and anatomical site classification of lesions [153]. The pathology foundation model BEPH, trained on large‐scale, unlabeled pathology image datasets, can learn clinically meaningful features for lung cancer classification and survival prediction. BEPH consistently outperformed existing models across multiple evaluation metrics, underscoring its promise for clinical deployment in lung cancer management [146].

LLMs can serve as virtual communication tools to provide clinical decision support and counseling for patients with lung cancer [154]. For instance, ChatGPT has demonstrated the ability to accurately respond to questions related to lung cancer prevention, screening, and common terminology found in radiology reports. This capability enhances patients’ understanding of their condition and empowers them to make informed decisions. ChatGPT can simplify technical jargon and deliver personalized explanations when confronted with complex medical information, facilitating patient comprehension and engagement [155, 156]. Moreover, LLMs have been shown to reduce anxiety in lung cancer patients by improving communication between patients and healthcare providers, thereby promoting better treatment adherence. In addition to strengthening patient‐provider interaction, LLMs enhance healthcare accessibility by offering convenient, on‐demand access to medical knowledge. As LLM technologies continue to evolve, their potential in the healthcare domain is expected to expand further, serving as practical tools for patient education and clinical decision‐making and ultimately bridging the gap between medical professionals and patients [157, 158].

### 5.3 Gastrointestinal Cancers

Generative AI have the potential to significantly enhance gastrointestinal (GI) cancer screening, particularly by increasing patient awareness of early detection. By generating easy‐to‐understand and accurate screening information, generative AI provide patients with actionable recommendations and promote the implementation of shared decision‐making in clinical practice, thereby improving the efficiency and effectiveness of GI cancer screening [159, 160]. Additionally, LLMs can accurately answer medical questions related to GI cancers and offer adequate emotional support, helping patients and caregivers better cope with the psychological burden of the disease [161]. Studies have shown that GPT‐4 performs with high accuracy, compliance, and reliability in recommending follow‐up and surveillance intervals after colonoscopy. The model effectively processes patient clinical data and delivers follow‐up suggestions that align closely with the U.S. Multi‐Society Task Force guidelines on colorectal cancer screening and surveillance [162].

LLMs can support the subtype classification of GI cancers, offering valuable insights for clinical decision‐making and patient management. Studies have demonstrated that by leveraging in‐context learning techniques, the GPT‐4V model can efficiently and accurately classify histological subtypes of colorectal cancer, effectively distinguishing tumor tissue from normal tissue. This approach is particularly advantageous under data‐scarce conditions, significantly improving the reliability and efficiency of medical image analysis [94]. GPT‐4 has also shown the ability to generate concise summaries of pancreatic ductal adenocarcinoma (PDAC) staging reports and classify tumor resectability. When employing a “chain‐of‐thought” (CoT) strategy, GPT‐4 achieved a resectability classification accuracy of 92%. This study highlights the substantial potential of LLMs in enhancing report standardization, improving physician‐patient communication, and increasing diagnostic efficiency [64].

LLMs are critical in clinical drug prediction and developing personalized treatment strategies. In the context of therapeutic prediction, LLMs assist in elucidating drug mechanisms of action (MOA) by aligning drug‐related biological processes and signaling pathways. This capability is particularly valuable in predicting drug sensitivity for cancers such as PDAC and esophagogastric adenocarcinoma, where LLMs can match drugs to MOA‐associated pathways, thereby providing more precise theoretical support for individualized treatment planning [126]. Moreover, CancerGPT has demonstrated the ability to overcome data scarcity through zero‐shot learning, successfully predicting synergistic drug combinations for rare cancer types such as pancreatic and liver cancer. These findings underscore the broad applicability of LLMs in biomedical research and their potential to advance precision oncology [124].

Foundation models have demonstrated tremendous potential in diagnosing and treating GI cancers. For GI malignancies, foundation models pretrained on pathological tissue images using self‐supervised learning can accurately predict patient survival outcomes, stratify patients into high‐ and low‐risk groups, and strongly support developing personalized treatment strategies [163]. The Virchow Foundation model, trained on pathological images from common and rare cancer types, enables pan‐cancer detection and biomarker prediction, including for colorectal and pancreatic cancers, with high accuracy, even on previously unseen datasets. Notably, the model can analyze routine hematoxylin and eosin (H&E) stained slides to predict cancer‐related biomarkers, thereby reducing reliance on additional testing and enhancing the efficiency of therapeutic decision‐making [76]. Similarly, when applied to pathological images of colorectal, gastric, and pancreatic cancers, the CHIEF model identifies cancerous cells with high precision and predicts molecular profiles based on cellular morphology. It can infer genomic alterations, identify DNA patterns associated with immunotherapy responses, and accurately estimate patient survival rates [77].

### 5.4 Other Cancer Types

When integrated with named entity recognition frameworks, LLMs can effectively extract key information from semi‐structured EHRs for thyroid cancer, enabling automated cancer staging and risk stratification. This significantly enhances clinical decision support. By employing a range of prompting strategies—including zero‐shot, CoT, and few‐shot learning—studies have shown that models such as Mistral, Gemma, LLaMA, and Qwen achieve high accuracy in thyroid cancer staging and risk assessment. Moreover, ensemble models have further improved the efficiency of staging and classification, highlighting the broad applicability of LLMs in clinical decision‐making for thyroid cancer [164].

Generative AI can interpret various types of medical imaging data, including X‐rays, CT scans, laryngoscopic images, and pathological slides. Studies have shown that commercial models, such as Claude 3.5 Sonnet and GPT‐4o, demonstrate superior performance, with Claude 3.5 Sonnet achieving an overall accuracy of 79.43%, outperforming open‐source models like HuatuoGPT‐Vision and LLaVA‐Med. However, performance varies across different imaging modalities, with notable discrepancies observed in interpreting laryngoscopic images and X‐rays. These findings highlight the need for further optimization of generative AI to enhance their accuracy in specific imaging domains. Overall, the results suggest that generative AI hold significant potential as clinical decision‐support tools, particularly in assisting decision‐making across various stages of laryngeal cancer surgery [165].

LLMs have demonstrated promising potential in the automated calculation of the Ovarian‐Adnexal Reporting and Data System (O‐RADS) MRI scores. A recent study evaluated two LLM‐based strategies: an LLM‐only approach and a hybrid strategy that combines feature‐based classification with deterministic rules. The results revealed that the hybrid strategy achieved significantly higher accuracy (97%) than the LLM‐only model (90%). Moreover, the hybrid model outperformed the original radiologist reports in terms of accuracy (97 vs. 88%). These findings suggest that optimized LLMs, when integrated with feature classification and deterministic rules, can effectively compute O‐RADS MRI scores, enhance the clinical utility of the O‐RADS system, reduce radiologist errors, and provide more accurate support for the diagnosis of ovarian cancer [166].

SkinGPT‐4, a pretrained multimodal LLM, has demonstrated remarkable progress in dermatology. Integrating a vision transformer with the Llama‐2‐13 b‐chat model allows the system to automatically diagnose a wide range of common skin conditions, including basal cell carcinoma, skin cancer, and hemangiomas, while providing interactive treatment recommendations. Studies have shown that SkinGPT‐4 achieves diagnostic accuracy comparable to that of board‐certified dermatologists. Its potential is particularly significant in resource‐limited settings, where access to specialized care is scarce. Through patient interaction, SkinGPT‐4 enhances communication efficiency, helps patients better understand their conditions, and offers timely and reliable therapeutic guidance—thereby advancing the precision of dermatological diagnosis and treatment [52].

## 6 Generative AI and LLMs in Cancer Clinical Trials

Clinical trials are critical in developing new therapies; however, insufficient patient recruitment frequently leads to delays or even trial termination [167, 168]. Traditional manual screening methods are time‐consuming, labor‐intensive, and resource‐intensive, substantially increasing the cost of trials [169, 170]. Although structured EHR queries have improved screening efficiency to some extent, they still rely heavily on manual interpretation of unstructured data, which limits overall performance [171]. In real‐world clinical settings, models often encounter complex cases or nonstandard terminology beyond their training corpus, resulting in performance degradation. With the rapid advancement of generative AI, their powerful NLP capabilities are driving transformative changes in clinical trials. Generative AI can efficiently parse unstructured data such as clinical narratives, imaging reports, and physician notes, enabling more accurate patient‐trial matching [172]. In addition, these models can assist in generating clinical trial protocols, automate data extraction and analysis, and support outcome prediction [173]. These technologies’ integration significantly enhances screening and recruitment efficiency and reduces labor costs, accelerating clinical trial progress [174]. Furthermore, digital twin technologies—integrating structured and free‐text data from EHRs—enable the construction of individualized patient timelines. These digital representations can simulate future health trajectories and assess the long‐term impact of interventions and comorbidities [175].

Patient‐to‐trial matching tasks can be categorized into two directional approaches: “trial‐to‐patient,” which matches a given clinical trial to a list of candidate patients, and “patient‐to‐trial,” which identifies a list of eligible clinical trials for a specific patient. For instance, the TrialGPT system adopts a patient‐to‐trial intelligent matching strategy, employing a three‐stage optimization pipeline to achieve efficient and accurate results. First, it performs intelligent retrieval of potential trials based on patient characteristics; second, it executes standardized fine‐grained matching; and finally, it generates a personalized ranking of recommended trials. Clinical validation demonstrates that TrialGPT can cover over 90% of relevant trials by screening only 5.5% of the trial database, achieving expert‐level matching accuracy and improving overall efficiency by 42.6%. LLMs significantly improve the efficiency and accuracy of clinical trial matching and enhance trustworthiness through transparent matching mechanisms, offering robust technical support for clinical trial processes [176]. Building on this foundation, the OncoLLM model further refines the workflow through its novel PRISM pipeline, which enables bidirectional intelligent search—matching patients to the most suitable trials and trials to eligible patients. In addition, the system supports continuous tracking, automatically identifying unmatched patients and recommending potential future trial opportunities. This dynamic matching mechanism substantially increases both recruitment rates and trial completion rates [177]. Architecturally, both TrialGPT and OncoLLM widely adopt dual‐encoder frameworks and dense passage retrieval to enhance the semantic matching of unstructured information. To meet the specific demands of clinical tasks, the models also incorporate multimodal contrastive learning during pretraining, thereby improving their capacity for multimodal information comprehension and contextual adaptation [176, 177].

Beyond patient matching, generative AI also demonstrate significant potential in the design phase of clinical trials. Data indicate that more than 40% of clinical trials suffer from design flaws, such as inadequate blinding, insufficient sample sizes, or suboptimal statistical plans, which critically undermine the scientific validity and reliability of trial outcomes. Generative AI, particularly application‐specific language models (ASLMs) tailored for clinical trial tasks, offers a novel solution to improve trial design quality. ASLMs can be developed through fine‐tuning general‐purpose models, integrating retrieval‐augmented generation (RAG) mechanisms, or training lightweight domain‐specific models. These approaches enhance domain adaptability, controllability, and data privacy. ASLMs assist in standardizing trial procedures and efficiently integrating external, high‐quality structured knowledge bases, thereby improving decision accuracy and regulatory compliance [174].

LLMs can extract key variables such as disease progression, adverse events, and treatment responses from heterogeneous data sources [178]. This capability supports more personalized and data‐driven trial designs. In addition, LLMs are increasingly applied in automated data reporting and adverse event monitoring, enabling real‐time risk detection and safety alerts through intelligent interpretation of clinical records and laboratory results [179, 180, 181, 182]. These applications significantly enhance the intelligence and regulatory efficiency of clinical trial workflows. Overall, LLMs are reshaping the paradigm of clinical trial design and execution, laying a robust technological foundation for developing more efficient, precise, and broadly representative clinical research systems. However, generalizability, interpretability, regulatory compliance, and privacy protection remain. Looking ahead, the emergence of ASLMs, the deep integration of multimodal data, and the advancement of model architectures with long‐term memory and causal reasoning capabilities will pave the way for end‐to‐end, fully integrated systems, spanning trial design, execution, and intelligent monitoring. These innovations are expected to accelerate precision medicine's translation and real‐world implementation (Table 3).

**TABLE 3:** Applications of generative AI in cancer clinical trials.

Clinical trial stage	Key tasks	LLM‐based approaches	Representative models/systems	Advantages	Challenges	References
Patient screening and recruitment	Identification of eligible patients	NLP‐based extraction of structured and unstructured EHR data	TrialGPT	Improves recruitment efficiency; reduces manual workload	Generalizability and data heterogeneity	[176]
Bidirectional patient‐trial matching	Patient‐to‐trial and trial‐to‐patient matching	Dual‐encoder framework; dense retrieval; multimodal contrastive learning	TrialGPT, OncoLLM	High accuracy and scalable matching	Interpretability and regulatory approval	[176, 177]
Dynamic matching and follow‐up	Continuous patient monitoring and rematching	Longitudinal patient modeling; digital twin integration	OncoLLM	Improves enrollment and completion rates	Data privacy and real‐time integration	[177]
Clinical trial design	Protocol generation; sample size estimation	Application‐specific language models (ASLMs); RAG‐based knowledge integration	ASLMs	Improves design quality and compliance	Validation and trustworthiness	[174]
Real‐world evidence integration	Extraction of disease progression, response, and outcomes	Multimodal data fusion from EHR, imaging, and pathology	Multimodal LLMs	Enhances generalizability and representativeness	Standardization and interoperability	[172]
Safety monitoring	Adverse event detection and pharmacovigilance	Automated NLP; signal detection	LLM‐based safety systems	Real‐time risk monitoring	False positives and clinical validation	[178]
Data reporting and regulatory support	Automated reporting and documentation	Structured report generation; compliance checks	Clinical NLP pipelines	Improves workflow efficiency	Regulatory and ethical challenges	[179, 180, 181]

## 7 Integration of Generative AI With Other AI Technologies

Multimodal medical data provide a robust foundation for the precise diagnosis and effective treatment of cancer [110]. Widely collected clinical data—including radiological imaging, histopathological slides, endoscopic videos, and dermatological images—play a critical role in early cancer screening and pathological confirmation, therapeutic decision‐making, and prognosis assessment [183]. Recent advances in vision–language pretraining models (e.g., BioMedCLIP, LLaVA‐Med) have achieved promising breakthroughs in joint image‐text modeling for oncology, demonstrating superior image‐text alignment and question‐answering capabilities. These developments lay the groundwork for multimodal decision support systems in cancer care. However, multimodal modeling still faces notable challenges. It heavily relies on high‐quality annotations and precise alignment across data modalities, while current models exhibit limited robustness to missing modalities and noise interference. Additionally, the high‐dimensional, sparse nature of omics data and limited sample sizes often result in overfitting and reduced generalizability. As medical imaging technologies continue to advance and their clinical adoption expands rapidly, the volume and complexity of data are growing exponentially, far exceeding the capacity for manual interpretation. This surge in data increases the cognitive burden on clinicians and raises the risk of misdiagnosis or missed diagnoses [184].

Against this backdrop, generative AI with strong cross‐modal understanding and knowledge integration capabilities are increasingly being applied to analyzing multimodal medical data [185]. Generative AI can effectively integrate heterogeneous data, such as medical images and clinical texts, to extract key diagnostic features, generate structured diagnostic reports, answer clinical queries, and support personalized treatment decision‐making [185]. In particular, LLMs built on deep learning architectures like Transformers have significantly improved the efficiency and accuracy of cancer image interpretation [94]. For example, in locally advanced gastric cancer, combining 3D modeling of preoperative CT scans with postoperative pathology enables early prediction of lymph node metastasis following neoadjuvant chemotherapy [186]. In breast cancer management, multimodal models incorporating histopathological slides and corresponding textual reports can accurately assess recurrence risk, thereby helping avoid overtreatment [187].

In addition to medical and pathological imaging, the rapid advancement of multiomics technologies—including genomics, transcriptomics, epigenomics, and single‐cell transcriptomics—has provided deeper molecular‐level insights into cancer mechanisms and personalized treatment strategies [188, 189, 190]. The DrBioRight 2.0 platform, built on LLMs, is designed to accelerate cancer research through large‐scale functional proteomics. By leveraging NLP, the platform enables users to intuitively explore and analyze complex datasets, facilitating the identification of biomarkers and therapeutic targets. A key innovation of DrBioRight 2.0 lies in its deep integration of LLMs with oncological omics data, which not only improves data accessibility but also accelerates the translation of research findings into clinical applications [119]. However, single‐omics approaches are insufficient to fully capture the complexity and heterogeneity of cancer. Multiomics data offer complementary biological perspectives, enabling a more comprehensive and dynamic tumor evolution and heterogeneity characterization. This lays a solid foundation for constructing systematic and panoramic cancer atlases [189, 190, 191]. Current research is progressively shifting from single‐omics analysis to integrated multiomics frameworks, aiming to explore intermodality interactions, reconstruct signaling pathways, and identify key driver factors. Despite their promise, multiomics datasets are often high‐dimensional, heterogeneous, and noisy, posing significant challenges for data integration and biological interpretation. In this context, LLMs are emerging as powerful tools for multiomics analysis [192, 193]. Generative AI can effectively integrate omics features, clinical phenotypes, and literature‐derived context to construct biologically meaningful pathway networks thanks to their knowledge reasoning, semantic extraction, and natural language generation capabilities. They can also predict drug sensitivity or therapeutic targets and generate natural language explanations and visualizations of model outputs [194, 195, 196]. This capability enhances the interpretability of computational models and facilitates the translation of bioinformatics discoveries into clinical practice.

Cancer research is evolving from static feature extraction to system‐level multimodal network integration, aiming to characterize the complex biological features of tumors more comprehensively [118]. Tumor progression typically involves multiscale and multilevel biological processes governed by highly intricate regulatory networks [197]. A significant challenge in precision oncology is integrating spatial transcriptomics, omics data, imaging features, and clinical narratives to construct dynamic cancer regulatory networks [198]. The focus of multimodal data fusion is shifting from simple data concatenation to deep semantic understanding and discovering biological causal relationships. In this transformation, generative AI with enhanced knowledge capabilities are poised to play a central role. Generative AI can efficiently integrate structured and unstructured information, supporting key tasks such as reconstructing cancer regulatory networks, identifying critical pathways, and predicting potential therapeutic targets. This marks a shift from conventional point‐to‐point prediction models toward system‐level comprehension, opening new avenues for intelligent, multimodal cancer analysis [94].

## 8 Challenges and Ethical Considerations

The performance of generative AI systems, including LLMs, diffusion models, GANs, and multimodal foundation models, is highly dependent on the quality of training data [199]. Low‐quality data—such as noise, annotation errors, or irrelevant content—diminishes training efficiency, impedes model convergence, and leads to misleading predictions in real‐world applications [200]. Such biases can have serious consequences in medical contexts, potentially compromising clinical decision‐making and patient safety [201]. A further concern is the phenomenon of “hallucination,” wherein generative AI generate content that is factually incorrect or entirely fabricated [202]. Similarly, image‐generative models may introduce unrealistic anatomical structures or synthetic artifacts that could affect downstream diagnostic performance. This poses substantial risks in healthcare, as it may result in inaccurate diagnostic suggestions, inappropriate treatment recommendations, or unreliable synthetic medical data [122]. Beyond data quality, deep learning methods face persistent challenges in reliability and interpretability. While adversarial training and knowledge distillation have been explored to enhance model robustness and deployment adaptability, their effectiveness in medical settings remains inconsistent and may even introduce new systemic biases. Common interpretability approaches—such as attention visualization or feature importance analysis—can improve model transparency to a certain extent. Yet, they often fail to elucidate the underlying causal mechanisms behind predictions. This limits their practical utility in high‐stakes clinical decision‐making. Data preprocessing is a critical step for ensuring model stability and predictive reliability. High‐quality datasets should involve rigorous data cleaning, denoising, and standardization protocols supplemented by expert medical annotation and sample curation. These practices are essential to maximize the model's generalizability and real‐world applicability [203].

Applying generative AI technologies in healthcare requires rigorous attention to data privacy, ethical governance, and responsible deployment [204]. Furthermore, generative AI models capable of producing synthetic images, clinical records, or virtual patient trajectories raise additional concerns regarding data ownership, informed consent, authenticity verification, and potential misuse of AI‐generated medical content. Patient health data are highly sensitive, and using such information for model training without explicit informed consent can lead to serious ethical controversies and a crisis of public trust [205]. Protective measures include data encryption, de‐identification, and access control to mitigate privacy risks. However, these approaches require careful calibration to balance privacy protection with maintaining model performance [105]. Additionally, the diverse and often inconsistent quality of LLM training data—some of which may be biased, outdated, or inaccurate—further compounds the risks associated with medical deployment [48]. Enhancing data transparency, establishing ethical review mechanisms, and implementing bias detection protocols are critical to ensuring LLMs’ trustworthiness and legal compliance in clinical settings [105]. Looking ahead, as multimodal medical datasets continue to expand and model complexity increases, regulatory strategies for LLMs will likely shift from static validation to dynamic monitoring. Privacy‐preserving techniques such as FL and differential privacy are expected to become increasingly integrated with model architectures, helping to safeguard data security while preserving computational efficiency. Ultimately, building an ethics governance framework aligned with the AI model lifecycle—featuring ongoing review, risk monitoring, and feedback loops—will be a foundational pillar for the sustainable development of intelligent healthcare systems.

Among the current challenges in translating generative AI technologies into oncology practice, clinical trust represents the most critical bottleneck, as it ultimately determines their adoption and sustainable integration into real‐world workflows. Although generative AI models demonstrate strong capabilities in diagnostic support and knowledge reasoning, their “black‐box” nature, reasoning variability, and occasional errors may undermine confidence among clinicians and patients [48]. These concerns are often rooted in upstream issues such as data quality and limited generalizability, which can lead to inconsistent performance across diverse clinical settings. Moreover, the clinical role of AI remains insufficiently defined. When model‐generated recommendations conflict with physicians’ judgment, uncertainties regarding liability, decision‐making authority, and professional responsibility frequently arise, particularly in high‐stakes oncology care. Therefore, future efforts should prioritize the development of trustworthy and clinically aligned AI systems by improving interpretability, uncertainty quantification, and transparency in human–AI collaboration. In parallel, standardized clinical evaluation frameworks and structured training programs are essential to enhance AI literacy and strengthen clinician confidence, thereby facilitating the responsible and sustainable integration of LLMs into oncology practice [206, 207].

**FIGURE 3:**
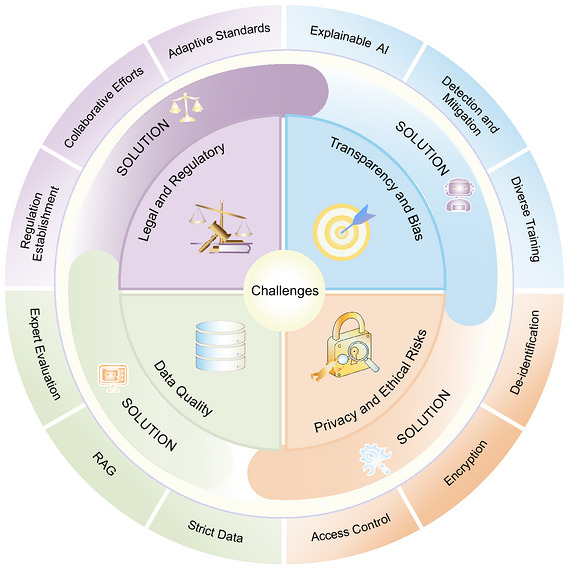
Key challenges and solutions for LLMs in medicine. This figure summarizes the major challenges in deploying LLMs in clinical oncology, including data quality, bias, privacy, and interpretability. These issues directly influence model reliability, fairness, and clinical trust. The proposed solutions—such as retrieval‐augmented generation, explainable AI, and privacy‐preserving learning—highlight the importance of hybrid and transparent model frameworks. Future regulatory strategies are expected to evolve toward adaptive and lifecycle‐based monitoring to ensure safety and accountability. *Abbreviations*: retrieval‐augmented generation (RAG).

Currently, the field of medical AI lacks unified and mature legal regulations and clinical validation frameworks, which significantly hinder the standardized deployment of generative AI in real‐world healthcare settings [208, 209, 210]. On the one hand, existing regulations do not comprehensively address full‐lifecycle governance, including model training, deployment, updating, and outcome feedback. On the other hand, model outputs' reliability, reproducibility, and safety still require systematic clinical trials and independent evaluations. In recent years, major countries and regions have begun to develop differentiated regulatory pathways for medical AI. The European Union has adopted a risk‐based framework through the Artificial Intelligence Act, emphasizing transparency, human oversight, and post‐market monitoring for high‐risk systems. In contrast, the United States, led by the Food and Drug Administration (FDA), has implemented a flexible, standards‐oriented approach that highlights total product lifecycle management, adaptive regulatory pathways, and prespecified change control plans to support continuous algorithm updates [211]. Meanwhile, China has established a rule‐based, data‐centric system focusing on algorithm traceability, data sufficiency, and multicenter validation, alongside dedicated governance policies for generative AI [212]. Despite these advances, significant gaps remain in cross‐border data governance, dynamic model updating, accountability, and the regulation of autonomous and multiagent systems. Therefore, internationally coordinated regulatory frameworks are urgently needed to standardize privacy protection, data governance, model validation, clinical performance evaluation, and responsibility boundaries across stakeholders [210]. Introducing third‐party certification mechanisms and clinical evaluation standards would enhance the adaptability and traceability of AI models in clinical environments, providing institutional safeguards for the compliant, secure, and efficient use of generative AI [213]. The US FDA has also emphasized that as AI technologies expand in healthcare, regulatory systems must remain adaptive to keep pace with rapid technological advancements, ensuring safety and efficacy. In particular, the rapid evolution of generative AI systems—including LLMs, multimodal foundation models, digital twins, and autonomous agents—introduces new uncertainties and regulatory challenges, underscoring the need for dedicated evaluation frameworks to ensure safety, reliability, and clinical effectiveness in oncology decision support [214, 215] (Figure 3).

## 9 Future Directions and Opportunities

By integrating vast volumes of medical literature, clinical data, patient histories, and real‐world evidence, generative AI are increasingly empowering key stages of clinical trials—including trial design optimization, patient recruitment, treatment pathway selection, and adverse event monitoring. Leveraging their advanced semantic understanding and information extraction capabilities, generative AI can dynamically identify critical insights from heterogeneous, multisource data, enabling personalized clinical trial design and execution. This significantly improves operational efficiency and optimizes treatment processes and outcomes through tailored patient management strategies, ultimately enhancing the overall patient experience. To realize these advancements, it is imperative to develop efficient data‐sharing platforms and intelligent patient management systems that support real‐time data updates and effective utilization.

With the continuous evolution of generative AI, foundation models are demonstrating increasing capabilities in multimodal fusion, causal reasoning, few‐shot learning, reinforcement learning, and autonomous decision support. For example, by integrating GNNs with medical knowledge graphs, generative AI can facilitate a causal understanding of cancer biology. When combined with RLHF, these models can continuously incorporate clinical insights to improve the accuracy and feasibility of personalized recommendations. Leveraging large‐scale data integration and contextual semantic modeling, generative AI hold the potential to uncover therapeutic strategies that are difficult to identify using traditional methods, thereby driving the emergence of a new paradigm in precision oncology. However, current generative AI still face challenges in generalizability, model update frequency, and clinical adaptability. To ensure their stable and safe deployment in real‐world settings, it is essential to establish cross‐sector and multidisciplinary collaboration mechanisms, strengthen regulatory and ethical oversight, and conduct systematic clinical validation.

The interpretability of generative AI is particularly critical in clinical practice. Future development should prioritize enhancing model transparency and comprehensibility, enabling clinicians to understand the reasoning processes and scientific rationale underlying the model's recommendations. This fosters greater trust in model outputs among healthcare professionals, improves the traceability of clinical decisions, and clarifies accountability. Moreover, incorporating individual patient characteristics—such as genetic background, lifestyle, and comorbidities—into personalized reasoning can significantly strengthen the model's precision in clinical decision‐making. To achieve this, developing AI algorithms with high interpretability and establishing closed‐loop clinical feedback mechanisms that continuously refine model performance and adaptability is essential. These efforts ensure generative AI practical utility and reliability in complex clinical settings.

Generative AI offer unique advantages in addressing the challenges of data scarcity and high heterogeneity commonly encountered in rare cancers and complex cases. By integrating global medical literature, rare case databases, and clinical data, generative AI can provide potentially viable treatment recommendations for patients lacking standardized therapeutic options. To effectively support such applications, it is essential to establish specialized, cross‐institutional data collection and sharing mechanisms that consolidate global healthcare resources and enable the development of precision case analysis platforms for rare diseases. Emerging technologies such as digital twins and generative AI models are also becoming key enablers in this context. Digital twins allow for the creation of virtual patient replicas to simulate disease progression and assess the outcomes of various intervention strategies. Diffusion models, conversely, can generate high‐quality synthetic data to augment limited real‐world samples, thereby enhancing the generalizability of generative AI under few‐shot conditions. The synergistic application of these technologies provides a robust data foundation and mechanistic insight for intelligent diagnosis and treatment of rare diseases, advancing the design, optimization, and validation of personalized therapeutic strategies into a new era.

The widespread application of generative AI in cancer research relies on continuous advancements in algorithmic capabilities and the deep integration of interdisciplinary collaboration and institutional support. Cooperation among biologists, clinicians, data scientists, and ethicists is essential to ensure these models’ safe, effective, and compliant deployment. Such collaboration also facilitates the development of unified technical protocols, ethical guidelines, and clinical integration frameworks. Future research should focus on building interdisciplinary R&D platforms that foster the convergence of innovative thinking, data resources, and technological pathways. This approach will support the high‐quality implementation of generative AI in cancer treatment. Additionally, generative AI evaluation should be incorporated into clinical standards to clearly define the model's role in therapeutic recommendations, delineate accountability, and establish mechanisms for ongoing assessment. These efforts are crucial for ensuring the stable integration and widespread adoption of generative AI within complex healthcare systems.

**FIGURE 4:**
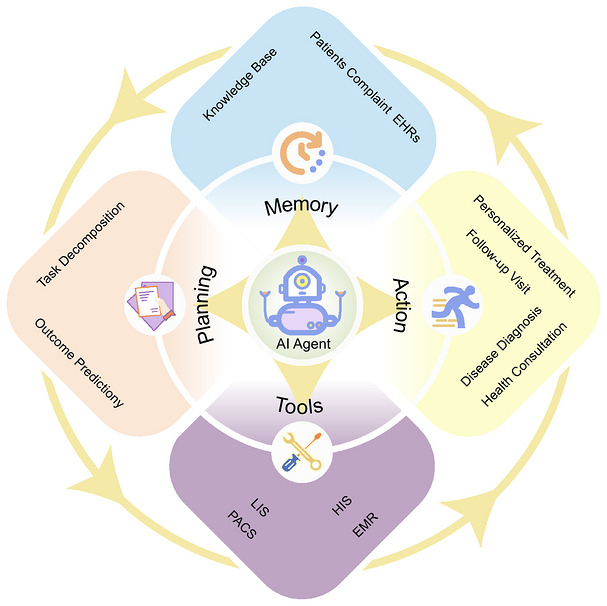
Future prospects of medical agents powered by LLMs. This figure presents an emerging paradigm in which LLMs function as the cognitive core of agent‐based intelligent oncology systems. By integrating memory, reasoning, and task execution, AI agents enable closed‐loop clinical workflows and dynamic treatment optimization. Such architectures support continuous monitoring, personalized intervention, and improved healthcare accessibility. *Abbreviations*: hospital information system (HIS); laboratory information system (LIS); picture archiving and communication system (PACS); electronic medical record (EMR).

The integration of AI agents is emerging as a key breakthrough to further enhance the practical value of generative AI in cancer research and treatment. As autonomous systems equipped with capabilities for perception, reasoning, and execution, AI agents can establish a closed‐loop “cognition–action” synergy with generative AI. In intelligent clinical scenarios, generative AI are responsible for deep comprehension of medical knowledge and strategy generation, while AI agents handle task execution, information retrieval, system interaction, and status monitoring. For instance, when a patient's condition or data status changes, agents can automatically initiate data synchronization, model invocation, and treatment plan generation, dynamically refining recommendations based on feedback. Different agents can take on specialized sub‐tasks within a multiagent architecture, such as health consultation, preliminary triage, diagnosis, treatment, and follow‐up. These agents operate in a shared semantic space with generative AI to collaboratively complete complex decision‐making workflows. This mechanism significantly advances the automation and personalization of cancer care, laying a technological foundation for end‐to‐end intelligent clinical pathway management systems. Looking ahead, the development of advanced agents with long‐term memory, reasoning, planning, and autonomous learning capabilities will enable deeper collaboration with generative AI, forming the core infrastructure of next‐generation intelligent oncology systems (Figure 4).

## 10 Conclusion

Generative AI is rapidly reshaping key aspects of cancer diagnosis and treatment. From multimodal data integration, synthetic data generation, and intelligent assisted diagnosis to personalized treatment planning, prognosis prediction, drug discovery, and clinical trial optimization, these technologies demonstrate strong capabilities in medical semantic understanding, knowledge reasoning, content generation, and predictive modeling, significantly accelerating the intelligent transformation of precision medicine. Open‐source models such as DeepSeek, Qwen, and Llama, together with emerging multimodal and generative frameworks, are increasingly applied in clinical text analysis, radiology and pathology report generation, biomarker prediction, drug recommendation, and patient management, forming an evolving ecosystem that connects data, models, and clinical scenarios.

Despite the remarkable progress achieved by generative AI and LLMs across multiple cancer types and clinical applications, important challenges remain regarding interpretability, reliability, data privacy protection, clinical adaptability, and generalizability. Future development should focus on three major directions: first, enhancing decision transparency and trustworthiness through multimodal semantic alignment, explainable AI, and causal reasoning; second, ensuring secure and compliant deployment in real‐world settings by integrating FL, differential privacy, knowledge distillation, and robust governance frameworks; and third, establishing closed‐loop human–AI collaborative systems powered by task‐oriented agents and multimodal foundation models to support the dynamic generation, execution, evaluation, and refinement of clinical strategies.

Furthermore, the convergence of LLMs with diffusion models, GANs, digital twins, multimodal foundation models, and multiagent architectures is expected to expand the application of generative AI in rare cancers, complex clinical scenarios, and highly personalized therapeutic pathways. Promoting interdisciplinary collaboration, establishing harmonized ethical and regulatory standards, and developing secure and interoperable medical data infrastructures will be essential for translating these technologies into routine clinical practice. Looking forward, future intelligent oncology systems will likely integrate generative AI as the foundational engine for knowledge generation and multimodal reasoning, with LLMs serving as the cognitive core and AI agents acting as the execution layer. Such systems have the potential to create a new generation of precision oncology ecosystems characterized by scalability, interpretability, adaptability, and continuous learning, ultimately transforming global cancer prevention, diagnosis, and treatment.

## Author Contributions

Yunfang Yu, Zhenhui Zhao, and Zehua Wang contributed to the conception of the review and drafted the manuscript. Yujie Tan, Ruichong Lin, Ting Li, and Daniel Baptista‐Hon participated in manuscript writing, literature organization, and critical revision. Yongjian Chen and Liancheng Yang were responsible for figure design, graphical abstract preparation, and data visualization. Man Tong and Lijun Zheng contributed to figure preparation and visualization. Xiaoxi Zhang and Chuan Wu critically revised the manuscript and provided important intellectual content. Kang Zhang, Junyan Wu, and Olivia Monteiro supervised the study, provided critical revisions and strategic guidance, and oversaw the overall writing process. All authors reviewed, edited, and approved the final version of the manuscript.

## Funding

This study was supported by grant from the National Natural Science Foundation of China (82404087), the GuangDong Basic and Applied Basic Research Foundation (No.2025A1515011563), the Guangdong Science and Technology Department (No. 2024B1212030002), the Scientific Research Launch Project of Sun Yat‐Sen Memorial Hospital (No. SYSQH‐II‐2024‐07), the Medical‐Engineering Integration Program at Sun Yat‐sen Memorial Hospital, Sun Yat‐sen University (No. YXYGRH202605), the Macau Science and Technology Development Fund, Macau (0003/2021/AKP), the Basic Research Project of the 2023 Guangzhou Science and Technology Plan Basic Research Program (City‐University (Institute)‐Enterprise Joint Funding for Dengfeng Hospital) (2023A03J0725), and the Fundo para o Desenvolvimento das Ciências e da Tecnologia (FDCT) (FDCT 0055/2022/A1 and 0022/2025/RIA1).

## Conflicts of Interest

Author Kang Zhang is an Editorial Board member of MedComm. Author Kang Zhang was not involved in the journal's review of or decisions related to this manuscript. Author Lijun Zheng is an employee in China United Network Communications Corporation but has no potential relevant financial or nonfinancial interests to disclose. The other authors declared no conflicts of interest.

## Ethics Statement

The authors have nothing to report.

## Data Availability

The authors have nothing to report.
